# Long-term outcomes following primary resection of intraosseous meningiomas with concurrent cranioplasty: a population-based Swedish multicentre study

**DOI:** 10.1007/s00701-026-06987-0

**Published:** 2026-07-18

**Authors:** Robert F. Nilsson, Alba Corell, Emilia Muncan, Peter Lindvall, Rickard L. Sjöberg, Richard Ågren, Bjartur Sæmundsson, Alexander Fletcher-Sandersjöö, Ulrik Birgersson, Jimmy Sundblom, Mats Ryttlefors, Göran Hesselager, David Fröjd Revesz, Teodor Svedung Wettervik, Klas Holmgren

**Affiliations:** 1 Department of Clinical Science - Neurosciences, Umeå University, 901 87 Umeå, Sweden; 2 Department of Clinical Neuroscience, Institute of Neuroscience and Physiology at the Sahlgrenska Academy, University of Gothenburg, Gothenburg, Sweden; 3 Department of Neurosurgery, Sahlgrenska University Hospital, Gothenburg, Sweden; 4 Department of Neurosurgery, Karolinska University Hospital, Stockholm, Sweden; 5 Department of Physiology and Pharmacology, Karolinska Institute, Stockholm, Sweden; 6 Department of Clinical Neuroscience, Karolinska Institute, Stockholm, Sweden; 7 Department of Medical Sciences, Section of Neurosurgery, Uppsala University, Uppsala, Sweden; 8 Department of Neurosurgery, Uppsala University Hospital, Uppsala, Sweden

**Keywords:** Intraosseous meningioma, Cranioplasty, Complications, Recurrence

## Abstract

**Background:**

Studies on intraosseous meningiomas requiring cranioplasty have been scarce, and surgical outcomes thus remain poorly characterized. Using a population-based, multicentre cohort of patients who had undergone resection of intraosseous meningioma with concurrent cranioplasty, we aimed to establish long-term outcomes, as well as rates and predictors of complications and tumour recurrence.

**Method:**

All patients who had undergone primary resection of an intraosseous meningioma with concurrent cranioplasty at four neurosurgical departments in Sweden between 2008 and 2022 were included. Data on patient demographics as well as tumour- and surgery-related data were collected retrospectively by scrutiny of medical records. Rates and predictors of surgical complications, and the composite outcome of tumour recurrence (including growth of residual tumour), were analysed using Cox regression.

**Results:**

Of the 119 patients included, 16% experienced surgical complications requiring reoperation, 10% underwent implant explantation, and 31% developed tumour recurrence. Choice of implant material differed significantly across the participating centres but did not influence complication rates. Markers of biological aggressiveness were associated with an increased risk of surgical complications and tumour recurrence.

**Conclusions:**

Surgical complications and tumour recurrence are common following resection of intraosseous meningiomas with concurrent cranioplasty. Rather than the material used for cranial reconstruction, biological and anatomical tumour-related factors were the strongest predictors of adverse outcomes.

**Supplementary Information:**

The online version contains supplementary material available at 10.1007/s00701-026-06987-0.

## Introduction

Intraosseous meningiomas may arise either as primary neoplasms of the cranial bones or secondarily through osseous extension of an intradural tumour [7]. They present a particular surgical challenge, as resection necessitates removal of the affected bone, for which clear tumour margins may be lacking [11]. While secondary hyperostosis may be managed by drilling of the hyperostotic bone to achieve adequate tumour removal, suspected tumour infiltration mandates craniectomy with subsequent cranioplasty [4]. Consequently, management of intraosseous meningiomas differs from that of exclusively intradural lesions due to the increased operative time, greater complexity associated with cranial reconstruction and – particularly in the skull base or orbital region – difficulty achieving a high extent of resection. In addition, primary intraosseous meningiomas more often exhibit higher malignancy grades [15] and are therefore more likely to require subsequent oncological treatment, primarily radiotherapy, with potential implications for skin integrity and long-term wound healing [26]. Furthermore, given the relatively low incidence of meningiomas with osseous involvement [16] that require concurrent cranioplasty, surgical outcomes, including complication rates, implant survival and tumour recurrence, remain poorly characterized. With few exceptions, available studies largely consist of case reports or small case series [4, 17], providing insufficient evidence to guide clinical decision-making. Notably, however, a substantial number of studies have evaluated cranioplasty and implant materials following decompressive craniectomy and found alarmingly high complication and reoperation rates [2, 12, 13, 23]. While these findings may not be directly extrapolated to cranioplasty performed in the setting of meningioma surgery, they suggest that a systematic evaluation of long-term outcomes in patients undergoing meningioma resection with cranial reconstruction has become highly warranted. To address these knowledge gaps, we conducted a near nation-wide, population-based, multicentre study to assess the long-term outcomes of cranioplasty performed concurrently with primary meningioma resection. Specifically, we aimed to evaluate clinical outcomes, as well as rates and predictors of complications and the composite outcome tumour recurrence (including progression of residual tumour), including whether or not the choice of implant material had an influence on these outcomes.

## Methods and materials

### Study design and patients

This is a population-based retrospective multicentre study, in which all patients who had undergone primary resection of a histopathologically verified intraosseous meningioma with concurrent cranioplasty between 1 January 2008 and 31 December 2022 at the neurosurgical departments in Stockholm, Uppsala, Umeå, and Gothenburg (2008 to 2018 exclusively) were eligible for inclusion. The study covers a catchment area of approximately 7.5 million people, corresponding to 72% of Sweden’s population.

### Data acquisition

Clinical and radiological data were retrieved by review of medical records during 2024 and 2025, allowing for at least one year follow-up. Demographic variables comprised age and sex, whereas tumour characteristics – including tumour location, maximal tumour diameter (mm), invasion of venous sinus or en-plaque growth pattern, Ki67-index, WHO-grade, tumour recurrence (including residual tumour regrowth) and time to recurrence – were recorded. Surgical variables of interest were Simpson grade, implant material used for cranial and dural reconstruction, respectively, operative duration, type of perioperative antibiotics, use of corticosteroids at the time of cranioplasty and type of closure material (skin staples or sutures). Synthetic cranioplasty materials were categorized into the following groups: polymethyl methacrylate (PMMA; also including titanium mesh reinforced with PMMA), plastic (porous polyethylene, polyetheretherketone), titanium-reinforced calcium phosphate (Ti-CAP), and titanium mesh. Clinical status pre- and postoperatively was assessed retrospectively using the modified Rankin Scale (mRS) [24] according to a prespecified study protocol designed to harmonize interpretations between assessors. Extent of comorbidities was quantified by means of the Charlson-comorbidity index [22] excluding age and supplemented with data on active smoking and diabetes. Type of craniectomy (uni- or bilateral) was registered, and the area of the cranial defect was calculated according to a previously described standardized method [20, 23]. Reoperation due to surgical complications (postoperative hematoma, infection, implant explantation, wound dehiscence, and bone resorption) or tumour recurrence was registered. Any chemo- or radiotherapy administered within 6 months of cranioplasty was also recorded. End of follow-up was defined as either the date when data was collected if the patient was still alive, the relocation date for patients who had moved to a different healthcare region during follow-up, or the date of death. Implant survival time was calculated based on the date of implant explantation or the end of follow-up.

### Statistical analyses

Continuous and ordinal variables were presented with medians and interquartile ranges (IQR), and categorical variables with frequencies and proportions. Ki67-index was categorized as low (< 5%), intermediate (5–10%), and high (> 10%), aligning with previous reports on tumour proliferation capability in meningiomas [25]. Associations between cranioplasty implant material and clinical or surgical variables were analysed using the Chi^2^-test, Kruskal–Wallis test, or Fisher’s exact test, as appropriate. Variables of interest were neurosurgical centre (Uppsala, Umeå, Stockholm, Gothenburg), tumour localization (skull base vs. non–skull base), craniectomy size, craniotomy laterality (uni- vs. bilateral), and occurrence of surgical complications. Hypothesized predictors for complications and tumour recurrence were selected á priori and included age, sex, Charlson-comorbidity index, diabetes, active smoking, use of corticosteroids, radiotherapy, chemotherapy, Ki67-index, WHO-grade, venous sinus infiltration, en plaque growth pattern, skull base localization, bilateral craniotomy, maximal tumour diameter, Simpson grade, craniotomy size, duration of surgery, closure material, implant material (with PMMA as reference group), and choice of antibiotic. Predictors were analysed with univariable Cox regression and, in case of statistically significant associations, included in a multivariable Cox regression analysis together with patient age. Multicollinearity among independent predictors was analysed with variance inflation factor (VIF). VIF > 5 was considered indicative of significant multicollinearity [14]. Implant survival and time to tumour recurrence and regrowth were illustrated using Kaplan–Meier graphs. For implant survival, type of perioperative antibiotic, corticosteroid treatment, WHO-grade, and Ki67-index, were selected as stratifying variables, whereas analyses of tumour recurrence were stratified for corticosteroid treatment, WHO-grade, Ki67-index, skull base localization, and Simpson grade. Censored observations represented patients without implant failure or tumour recurrence, respectively, at end of follow-up. Differences between survival curves were analysed with the log-rank test. All statistical analyses were conducted using the computer software Stata/BE version 17 (StataCorp LLC, TX, USA). Statistical significance level was set at *p* < 0.05.

## Results

### Patient, tumour and surgical characteristics

A total of 119 patients were included in this study. Patient characteristics are shown in Table 1, whereas tumour-related and surgical data are summarized in Table 2.

**Table 1:** Patient characteristics, *n* = 119

Variables	
Age (years), median (IQR)	56 (46–66)
Sex (male/female), *n* (%)	38/81 (32/68%)
Charlson-comorbidity index, median (IQR)	2 (2–2)
Diabetes (yes), *n* (%)	9 (8%)
Active smoking (yes), *n* (%)	13 (11%)
mRS preop, median (IQR)	1 (0–1)
mRS postop, median (IQR)	1 (0–1)
Time to postop mRS (months), median (IQR)	4 (3–6)
Corticosteroids at CP (yes), *n*(%)	76 (64%)
Radiotherapy < 6 months after CP (yes), *n*(%)	5 (4%)
Chemotherapy < 6 months after CP (yes), *n*(%)	0 (0%)
Follow up time after CP (months), median (IQR)	92 (42–136)

Abbreviations: *IQR* inter-quartile range, *mRS* modified Rankin scale, *CP* cranioplasty

**Table 2:** Tumour- and surgery-related characteristics, *n* = 119

Variables	
WHO-grade, n(%)	
1	103 (87%)
2	11 (9%)
3	4 (3%)
Missing	1 (1%)
Ki67 index, n(%)	
< 5%	61 (51%)
5–10%	25 (21%)
> 10%	10 (8%)
Missing	23 (19%)
Venous sinus invasion (yes), n(%)	47 (40%)
En plaque growth (yes), n(%)	53 (45%)
Local, n(%)	
Convexity	48 (40%)
Parasagittal	32 (27%)
Falx	13 (11%)
Sphenoid	24 (20%)
Skull base, other	2 (2%)
Max tumour diameter (mm), median (IQR)	50 (35–66)
Tumour recurrence or progression of residual tumour (yes), n(%)	37 (31%)
Time to recurrence (months), median (IQR)	22 (6–38)
Laterality of bone flap (uni-/bilateral), n(%)	72/47 (61/40%)
Simpson grade, median (IQR)	1 (1–4)
1, n(%)	64 (54%)
2, n(%)	10 (8%)
3, n(%)	4 (3%)
4, n(%)	41 (34%)
Craniectomy size (cm^2^), median (IQR)	37 (24–53)
Implant type (synthetic/autologous), n(%)	118/1 (99/1%)
Duraplasty material, n(%)	
Graft or synthetic	99 (83%)
Autologous	12 (10%)
None	8 (7%)
Cranioplasty material, n(%)	
PMMA	68 (58%)
Plastic	17 (14%)
Ti-CAP	17 (14%)
Titanium mesh	16 (14%)
Autologous	1 (1%)
Duration of surgery (min), median (IQR)	258 (176–247)
Closure material (sutures/staples), n (%)	34/84 (29/71)
Antibiotics, n (%)	
Cefuroxime	46 (39%)
Cloxacillin	61 (51%)
Clindamycin	8 (7%)
Erythromycin	1 (1%)
Missing	3 (3%)
30 day mortality, n(%)	2 (2%)

Abbreviations: *WHO* World Health Organization, *mm* millimetre, *IQR* inter-quartile range, *cm*^2^ square centimetres, *min* minutes

### Cranioplasty – type of implant and related complications

The choice of implant material differed significantly across the participating neurosurgical centres (*p* = 0.003). PMMA was the most commonly used material overall, although its frequency varied between centres, ranging from 83% in Gothenburg to around 50% in the remaining centres. Ti-CAP was relatively common in Uppsala and Stockholm (22% and 24% respectively) while no cases were observed in Gothenburg or Umeå. In addition, distribution of implant materials differed significantly between skull base and non-skull base tumours (*p* = 0.001) and between uni- and bilateral craniectomies (*p* = 0.026). Plastic and titanium mesh implants were more frequent among skull-base tumours, whereas PMMA and Ti-CAP were more common among non-skull base tumours. Differences in implant choice between uni- and bilateral craniectomies were most pronounced for Ti-CAP (7% vs. 26%) and titanium mesh (17% vs. 9%) implants (Table 3).

**Table 3:** Implant material versus neurosurgical center, tumour localization, and craniectomy size and laterality

	Obs, n	PMMA, *n* (%)	Plastic, *n* (%)	Ti-CAP, *n* (%)	Titanium mesh, *n* (%)	*p*
***Center***						**0.003**
Uppsala	37	20 (54%)	7 (19%)	8 (22%)	2 (5%)	
Umeå	26	13 (50%)	7 (27%)	0	6 (23%)	
Stockholm	37	20 (54%)	1 (3%)	9 (24%)	7 (19%)	
Gothenburg	18	15 (83%)	2 (11%)	0	1 (6%)	
***Local***						**0.001**
Skull base	26	10 (38%)	6 (23%)	1 (4%)	9 (35%)	
Non-skull base	92	58 (63%)	11 (12%)	16 (17%)	7 (8%)	
*Craniectomy size (cm*^*2*^*)*						0.191
Median (IQR)	118	39 (24–50)	40 (26–67)	37 (27–81)	27 (16–35)	
***Craniectomy laterality***						**0.026**
Unilateral	72	43 (60%)	12 (17%)	5 (7%)	12 (17%)	
Bilateral	46	25 (54%)	5 (11%)	12 (26%)	4 (9%)	

Abbreviations: *Obs* observations, *PMMA* polymethyl methacrylate, *Ti-CAP* titanium-reinforced calcium phosphate, *cm*^2^ square centimetres, *IQR* inter-quartile range

### Surgical complications and implant materials

Over the entire follow-up period, a total of 19 patients (16%) experienced at least one surgical complication requiring reoperation. Twelve patients (10%) underwent reoperation with implant explantation, 5 (4%) with implant repositioning, 7 (6%) due to postoperative hematoma, and 4 (3%) due to infection (the total number of distinct complications exceeds 19, as some patients [n = 9] experienced multiple complications simultaneously). Of the 19 patients who underwent reoperation, one required multiple returns to the operation theatre, while the remainder did not. No patients experienced wound dehiscence or resorption of autologous bone transplant. Implant type did not show any clear association with the occurrence of complications (Table 4).

**Table 4:** Surgical complications requiring reoperation stratified by implant material

	Obs, *n* (%)	PMMA, *n* (%)	Plastic, *n* (%)	Ti-CAP, *n* (%)	Titanium mesh, *n* (%)	*p*
Any complication	19 (16%)	14 (21%)	2 (12%)	1 (6%)	2 (13%)	0.519
Implant explantation	12 (10%)	9 (13%)	2 (12%)	0	1 (6%)	0.476
Hematoma	7 (6%)	5(7%)	0	1 (6%)	1 (6%)	0.871
Infection	4 (3%)	3 (4%)	1 (6%)	0	0	1.000
Reposition	5 (4%)	4 (6%)	1 (6%)	0	0	0.917
Wound dehiscence	0	0	0	0	0	N/A

Abbreviations: *Obs* observations, *N/A* not applicable (due to no observations), *PMMA* polymethyl methacrylate, *Ti-CAP* titanium-reinforced calcium phosphate

### Predictors of surgical complications and tumour recurrence

Table 5 depicts the univariable regression analysis for the hypothesized predictors of surgical complications requiring reoperation, implant explantation exclusively, and the composite outcome tumour recurrence (including residual tumour progression), respectively. Variables significantly associated with surgical complications (any kind) included corticosteroid treatment at the time of cranioplasty (HR 4.92, 95% CI 1.13–21.42, *p* = 0.034), higher Ki67-index category (HR 2.10, 95% CI 1.16–3.80, *p* = 0.015), higher WHO-grade (HR 2.15, 95% CI 1.13–4.10, *p* = 0.020), and greater maximal tumour diameter (mm) (HR 1.02, 95% CI 1.00–1.04, *p* = 0.033). When assessing implant explantation exclusively, statistically significant associations were observed for higher Ki67-index (HR 3.70, 95% CI 1.66–8.27, *p* = 0.001) and higher WHO-grade (HR 2.81, 95% CI 1.35–5.85, *p* = 0.006). Variables associated with tumour recurrence in univariable analyses included corticosteroid treatment (HR 2.77, 95% CI 1.21–6.34, *p* = 0.016), radiotherapy within 6 months of surgery (HR 7.95, 95% CI 2.65–23.90, *p* < 0.001), higher WHO-grade (HR 2.15, 95% CI 1.23–3.75, *p* = 0.007), skull base localization (HR 3.62, 95% CI 1.87–7.00, *p* < 0.001), larger maximal tumour diameter (HR 1.02, 95% CI 1.01–1.04, *p* = 0.005), higher Simpson grade (HR 1.59, 95% CI 1.26–2.02, *p* < 0.001), longer duration of surgery (HR 1.01, 95% CI 1.00–1.01, *p* = 0.002). Conversely, perioperative Cloxacillin use was associated with a decreased risk of recurrence (HR 0.51, 95% CI 0.26–0.99, *p* = 0.047).

**Table 5:** Variables associated with surgical complications (any kind), implant explantation specifically, and tumour recurrence, respectively (univariable Cox regression)

	Complication (any kind)		Implant explantation		Tumour recurrence or progression of residual tumour	
	HR (95% CI)	p	HR (95% CI)	p	HR (95% CI)	p
Age	0.99 (0.96—1.03)	0.734	0.98 (0.94—1.02)	0.319	0.98 (0.96—1.01)	0.145
Sex	0.93 (0.35—2.48)	0.888	0.92 (0.28—3.08)	0.899	0.82 (0.41—1.63)	0.567
Charlson-comorbidity index	1.05 (0.78—1.42)	0.744	1.18 (0.88—1.59)	0.278	1.09 (0.87—1.36)	0.474
Diabetes	1.42 (0.32—6.17)	0.644	2.25 (0.49—10.32)	0.296	2.18 (0.85—5.61)	0.107
Active smoking	0.43 (0.06—3.26)	0.416	N/E		0.61 (0.19—2.00)	0.417
Corticosteroids at CP	**4.92 (1.13—21.42)**	**0.034**	6.44 (0.83—49.89)	0.075	**2.77 (1.21—6.34)**	**0.016**
Radiotherapy	1.33 (0.17—10.17)	0.783	2.41 (0.30—19.56)	0.411	**7.95 (2.65—23.90)**	** < 0.001**
Ki67 index	**2.10 (1.16—3.80)**	**0.015**	**3.70 (1.66—8.27)**	**0.001**	1.41 (0.89—2.23)	0.144
WHO-grade	**2.15 (1.13—4.10)**	**0.020**	**2.81 (1.35—5.85)**	**0.006**	**2.15 (1.23—3.75)**	**0.007**
Venous sinus invasion	1.82 (0.71—4.62)	0.210	2.86 (0.86—9.56)	0.088	1.51 (0.79—2.91)	0.215
En plaque growth	0.46 (0.17—1.30)	0.145	0.62 (0.19—2.05)	0.432	1.56 (0.81—3.00)	0.186
Skull base localization	1.03 (0.34—3.13)	0.961	1.14 (0.31—4.20)	0.849	**3.62 (1.87—7.00)**	** < 0.001**
Bilateral craniectomy	0.93 (0.36—2.42)	0.889	1.48 (0.47—4.61)	0.502	0.60 (0.30—1.23)	0.165
Max tumour diameter (mm)	**1.02 (1.00—1.04)**	**0.033**	1.02 (1.00—1.04)	0.072	**1.02 (1.01—1.04)**	**0.005**
Simpson grade	0.88 (0.62—1.25)	0.493	0.97 (0.64—1.46)	0.872	**1.59 (1.26—2.02)**	** < 0.001**
Craniectomy size (cm^2^)	1.01 (0.99—1.03)	0.244	1.01 (0.99—1.03)	0.347	1.00 (0.98—1.01)	0.620
Duration of surgery (min)	1.00 (1.00—1.01)	0.818	1.00 (0.99—1.01)	0.631	**1.01 (1.00—1.01)**	**0.002**
Closure material (staples)	0.71 (0.27—1.91)	0.500	0.44 (0.14—1.41)	0.167	1.09 (0.51—2.31)	0.830
Plastic implant*	0.56 (0.13—2.48)	0.447	0.87 (0.19—4.07)	0.865	1.09 (0.44—2.69)	0.859
Ti-CAP implant*	0.30 (0.04—2.27)	0.243	N/E		0.57 (0.17—1.93)	0.368
Titanium mesh implant*	0.36 (0.05—2.75)	0.326	0.60 (0.08—4.74)	0.628	1.59 (0.64—3.93)	0.320
Cefuroxime	0.87 (0.32—2.32)	0.774	0.94 (0.28—3.15)	0.917	1.87 (0.97—3.59)	0.062
Cloxacillin	1.13 (0.45—2.87)	0.798	0.84 (0.27—2.62)	0.770	**0.51 (0.26—0.99)**	**0.047**
Other antibiotic	1.27 (0.29—5.61)	0.753	1.96 (0.42—9.18)	0,394	0.58 (0.14—2.41)	0.451

Abbreviations: *HR* hazard ratio, *N/E* not estimable, *CP* cranioplasty, *WHO* World Health Organization, *mm* millimetres, *cm*^2^ square centimetres, *min* minutes, *Ti-CAP* titanium-reinforced calcium phosphate

*PMMA set as reference group

In the multivariable Cox regression analysis, radiotherapy (HR 13.78, 95% CI 1.74–108.95, *p* = 0.013), skull base localization (HR 4.96, 95% CI 1.04–23.65, *p* = 0.044), and greater maximal tumour diameter (HR 1.03, 95% CI 1.01–1.06) remained significantly associated with tumour recurrence (Table 6). All included variables demonstrated VIF < 3 indicating limited multicollinearity (Supplementary Table 1 and 2).

**Table 6:** Variables independently associated with surgical complications (any kind) and tumour recurrence (including progression of residual tumour), respectively (multivariable Cox regression)

	Complication (any kind)		Tumour recurrence	
	HR (95% CI)	p	HR (95% CI)	p
Age	0.99 (0.96—1.03)	0.706	1.00 (0.96—1.05)	0.825
Corticosteroids at CP	1.75 (0.35—8.75)	0.493	1.49 (0.42—5.31)	0.538
Radiotherapy	-		**13.78 (1.74—108.95)**	**0.013**
Ki67 index	1.59 (0.72—3.52)	0.250	-	
WHO-grade	1.22 (0.45—3.35)	0.695	2.07 (0.74—5.80)	0.168
Skull base localization	-		**4.96 (1.04—23.65)**	**0.044**
Max tumour diameter (mm)	1.02 (0.99—1.04)	0.143	**1.03 (1.01—1.06)**	**0.013**
Simpson grade	-		0.88 (0.54—1.43)	0.601
Duration of surgery (min)	-		1.00 (1.00—1.01)	0.454
Cloxacillin	-		0.44 (0.11—1.80)	0.256

Hyphen indicates variables not included in the analysis. Abbreviations: *HR* hazard ratio, *CP* cranioplasty, *WHO* World Health Organization, *mm* millimetres, *min* minutes. VIF < 3 for all variables indicating limited multicollinearity

**Fig. 1:**
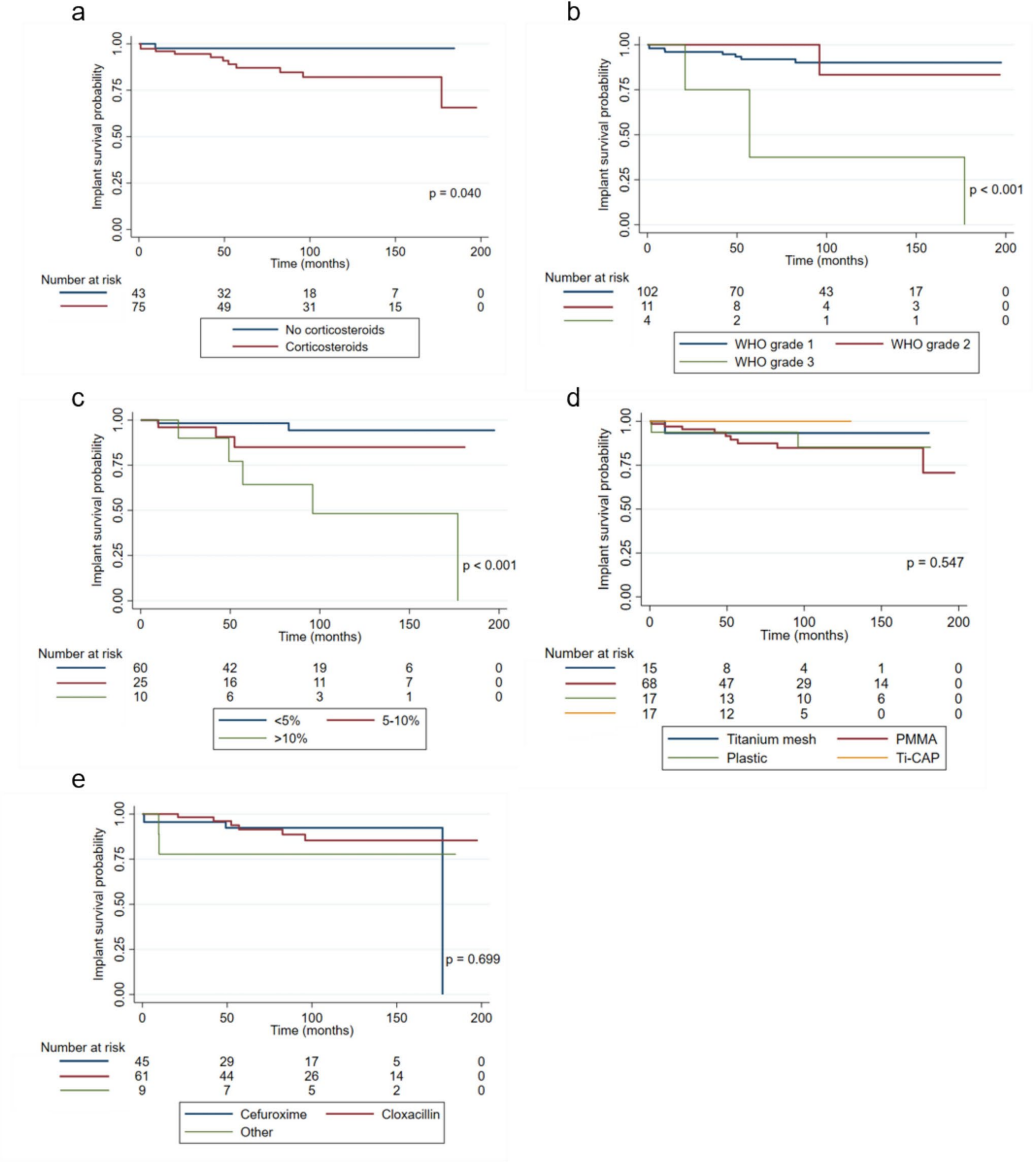
Kaplan–Meier plots depicting implant survival with corticosteroid treatment (**a**), WHO-grade (**b**), Ki67-index (**c**), implant material (**d**), and type of perioperative antibiotic (**e**) as stratifying variables

**Fig. 2:**
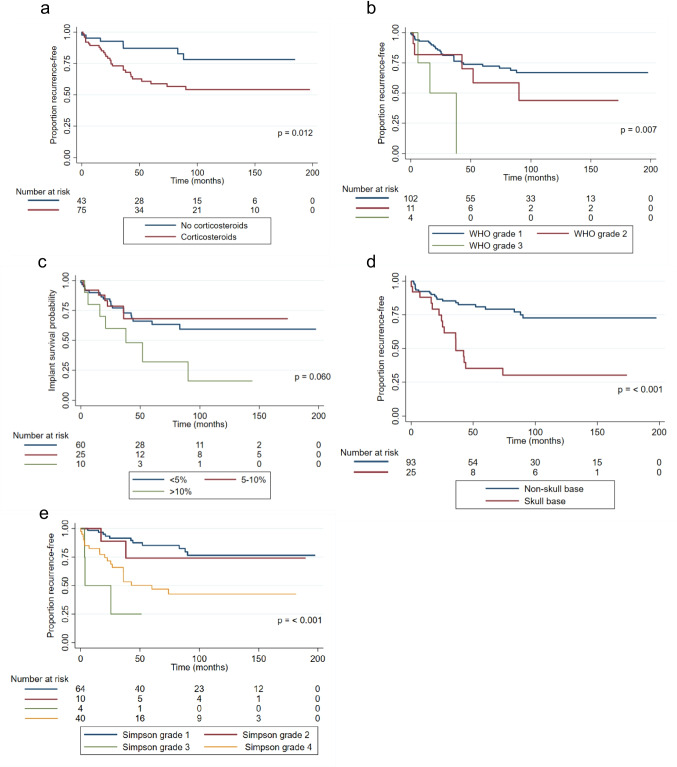
Kaplan–Meier plots depicting time to tumour recurrence or regrowth with corticosteroid treatment (**a**), WHO-grade (**b**), Ki67-index (**c**), skull base localization (**d**), and Simpson grade (**e**) as stratifying variables

Kaplan–Meier plots illustrating implant survival and time to tumour recurrence are provided in Figs. 1 and 2, respectively. Implant failure rates were significantly higher among patients receiving corticosteroid treatment at the time of cranioplasty (*p* = 0.040), and among patients with tumours displaying higher WHO-grade and Ki67-index, respectively (both *p* < 0.001). No statistically significant difference in implant survival was observed between types of implant material or perioperative antibiotics used. Time to recurrence was significantly shorter in patients treated with corticosteroids at the time of cranioplasty (*p* = 0.012), and in patients with tumours of higher WHO-grade (*p* = 0.007), skull base localization (*p* < 0.001), and higher Simpson grade of resection (*p* < 0.001). A trend towards a shorter time to recurrence was observed in tumours with a higher Ki67-index, however, statistical significance was not reached (*p* = 0.060).

## Discussion

In this near nation-wide Swedish multicentre study of 119 patients undergoing resection of intraosseous meningioma with concurrent cranioplasty, 16% experienced surgical complications requiring reoperation, and 10% underwent implant explantation, whereas tumour recurrence (including progression of residual tumour) was observed in 31% of cases. Distribution of implant materials differed significantly across the participating centres but did not appear to influence complication rates. Radiotherapy within 6 months of cranioplasty, skull base localization, and increased maximal tumour diameter were independently associated with tumour recurrence.

The overall rate of surgical complications aligns with previous studies on intraosseous tumour surgery, with the exception of postoperative infections, which were relatively low in the present cohort [4, 17]. The absence of wound dehiscence requiring reoperation was somewhat unexpected, particularly in light of prior reports indicating an increased risk of wound healing failure with synthetic implants compared to autologous cranioplasty [6]. One possible explanation may be the exclusion from our cohort of secondary cranioplasty cases (i.e., cranioplasty performed at different stage than the primary craniectomy). It is also notable that a recent study by our group on cranioplasty following decompressive craniectomy found no significant difference in wound dehiscence rates between autologous and synthetic implants, suggesting that the association reported in earlier studies may have been provisional [12]. As expected, the complication rate was higher compared with meningioma surgeries overall, most of which do not require cranial reconstruction [5, 21]. On the other hand, it was lower than figures reported for other cranioplasty populations, such as those undergoing reconstruction after decompressive craniectomy or surgical site infection [2, 12, 13, 23]. Several inherent differences in the surgical conditions might explain these observations. Similar to primary meningioma surgery, cranioplasty performed concomitantly with tumour resection represents a de novo surgical setting with generally good tissue quality, in contrast to isolated cranioplasty performed through a pre-existing scar where wound-healing conditions are often compromised. In addition, in meningioma surgery the scalp typically covers the same cranial volume before and after reconstruction. By contrast, patients with longstanding skull defects after decompressive craniectomy may develop a sinking skull flap and tissue contraction [12], complicating adaptation to the increased volume introduced by the implant, thereby increasing the risk for wound dehiscence. Of note, however, the anatomical aspects of cranial reconstruction in the setting of meningioma surgery differs substantially depending on tumour localization. For instance, tumours located over the convexity typically require a larger craniectomy and replacement implant, increasing the tension of the skin flap and potentially the risk for wound breakdown, as compared to skull base lesions. Notwithstanding, cranioplasty is arguably not considered the primary objective in meningioma surgery and may theoretically receive less focus compared with isolated cranioplasty procedures, particularly following long and technically demanding tumour resections. A key distinction from both other meningioma surgeries and many cases of isolated cranioplasty is the absence of autologous bone reconstructions, thereby eliminating the risk of bone flap resorption, which has been reported in up to 14% of cases [1]. Cranioplasty in the context of meningioma surgery is also inherently more heterogeneous in defect size and location compared with the standardized techniques used for decompressive craniectomy. This heterogeneity further underscores the limitations of prefabricated implants, as intraoperative findings may necessitate modification of the planned craniectomy. On the other hand, having a prefabricated implant may reduce operative times and yield better cosmetic results, compared to manually crafted implants. Finally, tumour-related factors make this type of cranioplasty unique, as adjuvant treatments such as corticosteroids, radiotherapy, or chemotherapy may influence wound healing and infection risk. Moreover, the potential for tumour recurrence and subsequent reoperation represents an additional long-term risk factor for complications independent of implant choice.

The notable differences in choice of implant material across the participating centres likely reflect local preferences and traditions, availability of materials, and institutional agreements with manufacturers. This heterogeneity underscores the absence of consensus guidelines for cranioplasty in the setting of intraosseous meningioma surgery. Instead, implant selection appears to be dependent on surgical anatomical factors. The association between implant choice and tumour location and craniectomy size is expected, as the flexibility of titanium mesh makes it suitable for irregular skull base defects, whereas PMMA and patient-specific implants are preferable for larger or more regular defects. The observed differences between unilateral and bilateral craniectomies, particularly for Ti-CAP implants, likely reflect similar practical considerations. Although the different implant materials evaluated in this study are representative of those most commonly used in Sweden over the past 15 years, some have since been discontinued or may not be commercially available in other countries. It is furthermore worth acknowledging that porous hydroxyapatite, a material widely used in several other European countries, has demonstrated superior performance to PMMA with respect to postoperative infection rates when employed following decompressive craniectomy [8, 9], and that no patient in the present cohort underwent reconstruction using this material.

Markers of higher malignancy grade were consistently associated with adverse surgical outcomes. Higher Ki67-index and WHO-grade predicted complications requiring reoperation, particularly implant explantation, and were also associated with shorter implant survival in the Kaplan–Meier analyses. Several mechanisms may contribute to these findings. High-grade intraosseous meningiomas more often exhibit aggressive behaviour, which may increase surgical complexity and thereby the risk of postoperative complications. These tumours also tend to cause more extensive peritumoral edema [19], making perioperative corticosteroid treatment more common, which in itself may predispose to wound-healing problems and infection [3]. Accordingly, corticosteroid use was associated with both complications and recurrence in the univariable analysis. Interestingly, however, Ki67-index was not associated with tumour recurrence in the regression analyses, whereas other markers of tumour aggressiveness, such as WHO-grade and radiotherapy, were. A possible explanation to this finding could be the relatively large number of missing values for Ki-67-index. Furthermore, Simpson grade showed a strong association with tumour recurrence, supporting previous evidence that radical resection remains one of the most important determinants of long-term tumour control [10]. It is also important to recognize that tumours exhibiting en plaque growth were prevalent (45%) in our cohort, which may have contributed to the high recurrence rate observed, as achieving a total or near-total extent of resection remains particularly challenging in these tumours. Likewise, skull base location, larger tumour diameter, and longer operative times likely act as proxies for increased surgical complexity and hence reduced ability to achieve radical resection, thereby explaining their associations with recurrence. Also of note, diabetes and smoking, despite their well-established association with complications [1, 12], did not emerge as significant predictors in the present study. The relatively low sample size and limited number of observations may however explain this finding.

In the multivariable analysis, no independent predictive factors for surgical complication were identified. For tumour recurrence, findings were largely consistent with the univariable analyses, reaffirming the influence of tumour biology and anatomical complexity on outcomes.

### Clinical implications

The findings of this study have several clinical implications. First, the strong influence of tumour biology on surgical complications and tumour recurrence highlights its importance in the risk assessment of these patients. Patients with markers of aggressive tumours may thereby benefit from closer perioperative surveillance and a lower threshold for early postoperative imaging and follow-up. Second, the lack of association between implant material and complication rates indicates that implant selection can likely be guided primarily by anatomical factors and surgeon preference, particularly in complex skull defects where reconstructive feasibility may outweigh material-specific considerations. Nonetheless, given the high incidence of recurrence and residual tumour regrowth in this patient population, the potential need for future reoperation should also be considered when selecting implant material, particularly in younger individuals and in cases with uncertain long-term tumour control. Third, the strong association between Simpson grade and recurrence reinforces that maximal safe resection remains a cornerstone of tumour control, not least in the context of intraosseous meningiomas. In cases where radical resection is not feasible and maximal safe removal is performed, early multidisciplinary discussion may be warranted, particularly for younger patients and those with high-grade tumours. Finally, the relatively high rates of recurrence and reoperation observed in this cohort emphasize the importance of structured long-term follow-up, including effective patient counselling regarding prognosis. It is striking that, although typically classified as benign, meningiomas can be associated with substantial surgical morbidity and risk of recurrence, particularly in those with intraosseous involvement. Tailored follow-up protocols based on biological aggressiveness and extent of resection may improve early detection of cranioplasty-related complications and recurrence, thereby optimizing long-term outcomes.

### Methodological considerations

The main strength of this study is its population-based design and near nation-wide coverage, ensuring high external validity and generalizability of the results. To our knowledge, it is the first multicentre study and largest cohort of patients undergoing resection of intraosseous meningioma with concurrent cranioplasty. In addition, it provides detailed clinical and radiological data for this scarcely studied patient population. However, several limitations must also be acknowledged. First, the observational study design may introduce bias and confounding. Second, data was obtained retrospectively, making it susceptible to imprecise assessments and inter-rater variability. However, a prior study has demonstrated high inter-rater reliability in retrospective assessments of performance status in patients with intracranial meningiomas [18]. Third, to facilitate statistical analyses, complication rates were analysed as binary outcome variables. Thus, patients who experienced multiple complications were not distinguished, which may have underestimated the effects of potential risk factors. Fourth, tumour recurrence and residual tumour regrowth were analysed as a composite measure. Although this might have obscured potential differences between these outcomes, it was considered reasonable given the limited sample size. Fifth, as numerous variables were evaluated across multiple outcomes, there is a risk of false-positive findings due to multiple testing, and p-values close to 0.05 should therefore be interpreted with caution. However, the study was exploratory in nature, and some variables were partly overlapping such as those related to malignancy grade, making adjustments potentially overly conservative. In addition, the number of variables included in the multivariable regression analyses was relatively large in relation to the number of events and the risk of overfitting should therefore be acknowledged.

## Conclusion

In this large, multicentre study of patients treated for intraosseous meningioma with concurrent cranioplasty, 16% experienced surgical complications resulting in reoperation, while nearly one-third of patients developed tumour recurrence or regrowth of residual tumour. Choice of implant material differed substantially between centres but did not appear to directly influence complication rates or implant survival. Instead, biological and anatomical tumour-related factors were the strongest predictors of adverse outcomes. Although these findings should be interpreted with caution in light of limited statistical power, they underscore the central role of tumour biology and anatomical considerations in guiding perioperative planning, risk assessments, and postoperative surveillance. Further prospective studies are warranted to refine treatment strategies and optimize long-term outcomes for this uncommon but challenging patient population.

## Supplementary Information

Below is the link to the electronic supplementary material.

Supplementary file1 (DOCX 16 KB)

## Data Availability

Upon reasonable request, data and methodology that support the findings of this study, including STATA software code, can be shared. However, access to such data will be subject to external review by the Swedish Ethical Review Board and Uppsala University concerning data sharing according to the European Union general data protection regulation (GDPR).

## Abbreviations

CI: Confidence interval
IQR: Interquartile range
Ki67: Ki-67 proliferation index
mm: Millimetres
mRS: Modified Rankin Scale
HR: Hazard ratio
PMMA: Polymethyl methacrylate
Ti-CAP: Titanium-reinforced calcium phosphate
VIF: Variance inflation factor
WHO: World Health Organization
