# Supplementary material for: Long-term outcomes following primary resection of intraosseous meningiomas with concurrent cranioplasty: a population-based Swedish multicentre study
Source: Acta Neurochir (Wien). 2026 Jul 18;168(1):160. doi: 10.1007/s00701-026-06987-0 (PMC13380628; doi:10.1007/s00701-026-06987-0)
Supplement: Supplementary file 1 — Supplementary file1 (DOCX 16 KB) [file 701_2026_6987_MOESM1_ESM.docx]

| Variable | VIF |
| --- | --- |
| Age | 1.10 |
| Corticosteroids at CP | 1.20 |
| Ki67 index | 1.60 |
| WHO-grade | 1.56 |
| Max tumour diameter (mm) | 1.14 |

**Supplementary table 1. Variance inflation factors for variables included in multivariable Cox regression analysis regarding implant explantation**

Abbreviations: VIF = variance inflation factor; CP = cranioplasty; WHO = World Health Organization; mm = millimetres.

**Supplementary table 2. Variance inflation factors for variables included in multivariable Cox regression analysis regarding tumour recurrence**

| Variable | VIF |
| --- | --- |
| Age | 1.21 |
| Corticosteroids at CP | 1.26 |
| Radiotherapy | 1.26 |
| WHO-grade | 1.30 |
| Skull base localization | 2.13 |
| Max tumour diameter (mm) | 1.71 |
| Simpson grade | 1.90 |
| Duration of surgery (min) | 2.38 |
| Cloxacillin | 1.37 |

Abbreviations: VIF = variance inflation factor; CP = cranioplasty; WHO = World Health Organization; mm = millimetres; min = minutes.
